# Apomorphia as an Emetic

**Published:** 1885-10

**Authors:** 


### Apomorphia as an Emetic.

If the reader will reflect for a moment, he will understand the value of such a remedy as apomorphia—a remedy that will cause prompt and rapid emesis when injected subcutaneously. Routh reports two cases that demonstrate its action very nicely. The first case was one of oxalic acid poisoning ; the patient, when found, was already dying ; the hands were clenched, froth and blood was oozing from her mouth, and respiration apparently had ceased. The pulse was barely perceptible. She had not vomited. 0.004 gramme

(gr. $\frac{1}{16}$) of apomorphia was now injected subcutaneously, and in $2\frac{1}{2}$ minutes the contents of the stomach were suddenly and forcibly ejected; the pulse became perceptible, but soon ceased entirely. The second case was of a woman found in a comatose condition, evidently due to spirits, since she was in the habit of taking large quantities of brandy. She could not be aroused. The pupils were dilated, pulse was intermittent, respiration stertorous, and the stomach full of fluid. 0.004 gramme of apomorphia was injected under the skin. The effect was to cause the emesis of one pint of alcoholic fluid in the course of $3\frac{1}{2}$ minutes, and in the course of 5 minutes more almost a quart of pure brandy was ejected. Pulse and respiration began to improve rapidly, and in twelve hours the patient awoke and felt good.

According to Routh, apomorphia does not excite vomiting during chloroform narcosis.—*Pharmaceutische Post.*

